# The UFair Questionnaire: Measuring Perceived University Unfairness and Its Association with Students’ Mental Health

**DOI:** 10.3390/bs15091280

**Published:** 2025-09-18

**Authors:** Raphael M. Herr, Veronika M. Deyerl, Katharina Diehl

**Affiliations:** Department of Medical Informatics, Biometry and Epidemiology, Friedrich-Alexander-Universität Erlangen-Nürnberg (FAU), 91054 Erlangen, Germany

**Keywords:** unfairness, injustice, questionnaire, students, university, stress, mental health, validation, organizational justice, Germany

## Abstract

University students face various stressors that may jeopardize their mental health. The aim of this study was to adapt the concept of organizational justice to universities, develop and validate a corresponding questionnaire (UFair: University Fairness Questionnaire) in German, and estimate its association with mental health. Perceived university unfairness was measured in 1105 students using the newly developed 20-item UFair Questionnaire. Mental health was assessed using the Patient Health Questionnaire (PHQ-8, depression) and the Maslach Burnout Inventory for Students (MBI-SS, burnout). The UFair Questionnaire had good psychometric properties, a unidimensional factor structure, and a considerable association with the mental health indicators. Perceived injustice also represents a stressor in the university setting, showing a considerable relation with students’ mental health. The relevance of these aspects to the health of students should be recognized and considered by universities. Valid measurement using the UFair Questionnaire represents the basis for possible preventive approaches and interventions.

## 1. Introduction

Mental health is becoming an increasingly important issue in modern society. The prevalence of mental diseases has increased in recent decades (World Health Organization, 2022). One major cause of mental disease is persistent stress (Marin et al., 2011). For instance, psychosocial work stressors are well-established risk factors for common mental disorders, such as depression and anxiety (Rugulies et al., 2023; Stansfeld & Candy, 2006).

A specific group that faces a variety of stressors potentially jeopardizing their mental health are university students. There is increasing evidence that stress during studies is associated with poorer learning outcomes, a lower quality of life, and poorer mental health outcomes, such as depression, anxiety, and burnout (Breiter et al., 2015; Brenneisen Mayer et al., 2016; Dahlin et al., 2005; El Ansari et al., 2014; Opoku-Acheampong et al., 2017). However, the primary focus of previous research was on students’ general perception of stress, which is not directly related to the university setting (e.g., Moffat et al., 2004).

To draw conclusions regarding preventive measures, it appears helpful to investigate stress directly caused by studying (Dyrbye et al., 2006; Heinen et al., 2017). In addition, it might be worthwhile to have a theoretical foundation that is directly linked to the target group of university students to derive insights. One possible solution to measure the direct stress caused by studying is to adapt psychosocial work stress models to the university setting.

First research in this regard has been conducted by measuring the psychosocial stress caused by studying in German university students based on the Effort–Reward Imbalance (ERI) model (Siegrist, 1996). For this purpose, the so-called ERI-student Questionnaire, which contains an adaptation of the original ERI Questionnaire to the target group of students, was used (Wege et al., 2017). It has been shown that all theoretically predicted components of the model, such as high effort in combination with low reward (the so-called effort–reward imbalance), were significantly associated with both poorer self-reported health and symptoms of depression and anxiety (Hilger-Kolb et al., 2018).

Another stress-theoretical construct established in the work context is the organizational justice model (Colquitt et al., 2001). Organizational justice is a stress model complementary to the ERI model, which captures the extent of perceived unfair treatment in the workplace. The association between perceived unfairness and increased stress is supported by various theories. For example, the deontic model of justice describes that perceived unfairness represents a violation of the fairness norm as a social norm and therefore leads to negative emotions (Folger & Skarlicki, 2008). However, a lack of fairness can also lead to stress, as it highlights that job demands exceed individual coping resources (Fischer et al., 2014). Additionally, a gap between one’s capabilities and the work demands becomes apparent (Vermunt & Steensma, 2003).

The lack of organizational justice at the workplace has been shown to be associated with negative health outcomes, including physical and mental health (Ndjaboue et al., 2012; Robbins et al., 2012; Scalabrin et al., 2022). It is conceivable that organizational injustice also exists within the university context and is associated with the health of students (Herr et al., 2022). Adapting this established model to the university setting can shed light on stressful psychosocial aspects of studying that have not been considered and investigated before.

Consequently, the aim of this mixed methods project was to adapt the concept of organizational justice to the university setting by developing and validating a corresponding Questionnaire (“UFair”: University Fairness Questionnaire) (Herr et al., 2022). In the first step, aspects of unfairness in the university perceived by students were determined through a qualitative study (Herr et al., 2024). Various sources of injustice perceived by students in the university setting could be identified (Diehl et al., 2024). These included organizational aspects such as enrolment and administrative processes, the treatment of students, including the behavioral patterns of lecturers and university staff, and the individual situation of students. In terms of the individual situation, international students and students from lower socio-economic backgrounds were identified as being particularly affected by unfairness. Based on these findings, theoretical considerations, and previous research, 60 items were developed and pre-tested in advance of the study presented here. A cognitive pretest (n = 95) was conducted including psychometric analyses, such as methods of classical (e.g., item-total correlation and Cronbach’s alpha [internal consistency] for reliability and exploratory factor analysis for validity) and probabilistic (e.g., item response theory) test theory. In addition, expert interviews were carried out (content validity). After these comprehensive pretests, the UFair Questionnaire consisted of 20 items used in a nationwide survey.

The aim of this manuscript is to report the quantitative validation of the UFair Questionnaire based on a nationwide sample of university students in Germany. In addition, its independent association with health outcomes, such as depression and burnout, will be presented. Furthermore, a focus is placed on international students and students with different socio-economic backgrounds.

## 2. Materials and Methods

### 2.1. Study Participants

This study included data form 1105 students who participated in an online survey. The sample was quoted according to the actual distribution of gender (approximately 50% female and male), and type of institution of higher education (60% university, 40% university of applied sciences) in Germany. Participants were funded and invited from a panel of a market research institute. All participants gave informed consent and the local ethic commission approved the study (Ethic committee II of the University of Heidelberg, 2019-1123N, 31 January 2019/19 October 2020).

### 2.2. Measurements

*UFair Questionnaire.* All students rated the 20 items of the UFair Questionnaire on a 5-point Likert scale from 1 = “does not apply at all” to 5 = “applies completely” (see Table 1). The mean score was calculated, with higher values indicating a greater extent of perceived unfairness.

*Depression.* Depression was measured with the German version of the Patient Health Questionnaire (PHQ-8) (Kroenke et al., 2009). The Cronbach’s α of the Questionnaire was 0.882. A mean score was calculated ranging from 1 to 4 with higher values indicating more depressive symptoms.

*Burnout.* Burnout was measured by the German version of the Maslach Burnout Inventory for Students (MBI-SS) (Gumz et al., 2013; Maslach et al., 1997). The Cronbach’s α of the 15 items was 0.841. The calculated mean score ranged from 1 to 7, with higher values indicating higher burnout.

*International students.* International students were identified if they answered the question: “Are you currently completing a semester/year abroad at a German university?” with yes.

*Subjective Social Status (SSS).* The Subjective Social Status was measured with the MacArthur Scale (Adler et al., 2000). This scale is a single-item measure in which students assesses their perceived rank relative to others in their group on a ladder with 10 rungs (Diehl et al., 2017). The students were split into tertiles with low, middle, and high SSS.

*Control variables.* Gender was classified as male or female (no diverse persons participated in the study). Age was assessed as a continuous variable. The type of institution of higher education was determined by the categories: university or university of applied sciences. Lifestyle factors were assessed as frequency of alcohol consumption (never; once a month or less; two to four times a month; two to three times a week; four or more times a week), smoking habits (yes, daily; yes, occasionally; no, not anymore; never smoked), physical activity (no physical activity; less than 1 h a week; regularly 1 to 2.5 h a week; more than 2.5 h a week), and nutrition (number of portions of fruit and vegetables per day: 0–2 portions, 2–4 portions, 5 or more portions).

### 2.3. Statistical Analyses

Validity of the new UFair Questionnaire was assessed by item analyses (mean values and standard deviation), internal consistency (Cronbach’s α, item-total correlation, and α if item is deleted), and structural validity (exploratory factor analysis [EFA] with principal component extraction and confirmatory factor analysis [CFA]). Linear regression models estimated the association between perceived university unfairness and the mental health indicators depression and burnout. The models were unadjusted (Model I), and adjusted for age, gender, type of university and lifestyle factors: alcohol consumption, smoking habits, nutrition, and physical activity. In addition, analyses were stratified for international students (yes vs. no), and the SSS groups (low, middle, high). Mean differences in the UFair Questionnaire were analyzed by T-Tests (two groups), and Analysis of Variance (ANOVA) with Post Hoc comparison and Bonferroni correction (three groups). All analyses were performed with IBM SPSS Statistics version 29, except the CFA that was conducted with R 4.5.1 (lavaan package (Rosseel, 2012)). Level of significance was set at *p* < 0.05.

## 3. Results

The participant characteristics are presented in Table 2. As described earlier, gender was almost equally divided into men and women (female: 50.14%, male: 49.86%). The average age was 25.5 years (SD = 5.43), and most students studied at universities (67.24% vs. 32.76% at university of applied sciences). The largest proportion never smoked (60.58%), consumed alcohol two to four times a month (34.22%), was 1 to 2.5 h physical active a week (37.47%), and ate 0 to 2 portions of fruits and vegetables per day (47.07%). 15.51% were classified as international students.

The corrected item-total correlation was between 0.650 and 0.794, with a Cronbach’s α of 0.960 (for not international students α = 0.954, international students α = 0.968; for low SES α = 0.950, middle SES α = 0.960, high SES α = 0.966), which did not improve after deletion of specific items (Table 1). The EFA extracted one component explaining 57.11% of the variance (Kaiser–Meyer–Olkin [KMO] = 0.981). The factor loadings ranged between 0.682 and 0.821. An additional confirmatory factor analysis (CFA) conducted for sensitivity reasons demonstrated an excellent (Comparative Fit Index [CFI] = 0.953; Tucker–Lewis Index [TLI] = 0.948; SRMR = 0.031) to acceptable (RMSEA = 0.059) fit.

The mean of the UFair Questionnaire was 2.58 (SD = 0.89) in the total sample (Table 3). International students had significant higher UFair mean values than non-international students (3.14 [SD = 1.07] vs. 2.47 [SD = 0.81], *p* < 0.001). The mean values for the SSS groups did not differ significantly (*p* > 0.05), with the middle group had the lowest values (2.46, SD = 0.83), followed by the low SSS group (2.59, SD = 0.81), and somewhat higher values in the high SSS group (2.62, SD = 0.97).

The UFair Questionnaire showed a considerable association with depression (beta = 0.473, *p* < 0.001, R^2^ = 0.224, Model I, Table 4), and burnout (beta = 0.467, *p* < 0.001, R^2^ = 0.218) in the total sample. The associations became even stronger after controlling for age, gender, type of institution of higher education, and lifestyle factors: depression beta = 0.485 (*p* < 0.001, R^2^ = 0.261, Model II, Table 4), burnout beta = 0.481 (*p* < 0.001, R^2^ = 0.250). The associations were especially pronounced for international students (depression: beta = 0.731, *p* < 0.001, R^2^ = 0.579; burnout: beta = 0.547, *p* < 0.001, R^2^ = 0.439, Model II, Table 4), and students in the high SSS group (depression: beta = 0.598, *p* < 0.001, R^2^ = 0.371; burnout: beta = 0.535, *p* < 0.001, R^2^ = 0.311, Model II, Table 4).

## 4. Discussion

In this study, we could confirm the validity of the new UFair Questionnaire using item analyses, internal consistency, and structural validity. In addition, a profound independent association was found between the UFair Questionnaire and mental health indicators. The UFair Questionnaire revealed a remarkable association not only with an indicator for health outcome that was adapted to the university setting (Maslach Burnout Inventory for Students) but also with a general health outcome (i.e., general depressive symptoms), indicating that the UFair Questionnaire measure is not limited to the variance that is attributable to the university setting. Thus, the UFair Questionnaire represents a valid tool for assessing the perceived injustice among university students.

The UFair Questionnaire revealed that German students perceive injustice in the university setting. Highest mean values were found for items describing that students’ concerns are difficult to address, participation in decision making is limited, lack of transparency, and lack of prompt and comprehensive information. These mainly describe structural issues that could be solved in cooperation among students, student councils, and university administration. It is conceivable to foster shared decision making, to make university structures and processes more transparent to students, and to focus more on the communication between administration and lectures and students. According to the quality criteria for a health-promoting university, it should operate with transparent communication and information management systems, and actively promote equal opportunities (cf. Bonse-Rohmann, 2023).

In addition to its use in scientific research, the UFair questionnaire could be used at individual universities to assess the current status of students’ perceived unfairness. Universities could gather valuable data by incorporating the UFair Questionnaire in routine internal student surveys. The findings could then inform local interventions.

Our findings on the relationship between perceived unfairness and mental health agree with those of previous research. A study exploring the association of university unfairness with burnout in Spanish students reported a correlation of 0.79 (Navarro-Abal et al., 2018). This study, however, used an organizational questionnaire for the workplace and not a questionnaire specifically adapted to and developed for the university setting. Nonetheless, it underlines the importance of considering perceived justice regarding preventing mental health problems in students.

In our analyses, we identified subgroup differences. First, the associations were especially pronounced for international students. This is in line with previous findings, which showed that perceived racial discrimination, which international students experience more frequently than the majority group of students, is associated with several negative mental health outcomes (Hwang & Goto, 2009). Secondly, the strength of association between perceived unfairness and mental health outcomes differed depending on students’ SSS. Surprisingly, the strongest relations were found in the group of students with high SSS. Previous research showed contradicting findings by identifying students from less advantaged backgrounds as more vulnerable for certain mental health problems compared to students with higher social status (Ibrahim et al., 2013; Steptoe et al., 2007). However, these studies did not consider self-perceived social status, which might be responsible for the differences found. Another possible explanation for the presented finding could be that students with a high SSS may experience less injustice in other areas of their lives, as they may be more privileged than those with a low SSS. Consequently, they may be less accustomed to unjust structures and therefore less resilient and have fewer coping strategies to deal with unfairness (Hu et al., 2016). This could result in a higher negative impact of perceived unfairness in the university setting on their mental health. According to the group-value model, fairness communicates two symbolic messages regarding membership. First, whether the individuals are respected members of their group and, second, whether the members could be proud of the group as a whole (Lind & Tyler, 1988; Tyler et al., 1996). Thus, fairness implies that students are respected members of a respected group. In contrast, unfairness refers to a marginal social status (Tyler & Smith, 1998). Therefore, it can be speculated that a low(er) social status, as indicated by unfairness, represents a greater threat to students with a high SSS. As a consequence, they experience more stress and might report worth mental health and the connection between unfairness and mental health is therefore more pronounced for them. However, this hypothesis needs to be confirmed through further research.

This study has some limitations that should be considered when interpreting our findings. First, this study was limited to students in Germany. Since university settings, and thus possibly the extent of perceived injustice within them, often differ between countries, future research focusing on countries other than Germany or country comparisons should be considered. Second, because our study was conducted in Germany, we only validated the German version of the UFair Questionnaire. It would be important to validate other versions in other countries. Third, the collected data relies on students’ self-reports. Consequently, a bias in the results due to social desirability could exist. Third, due to the cross-sectional study design, no causal order could be established between perceived unfairness and mental health outcomes. Hence, it is possible that initial mental health problems may have an impact on perceived unfairness in the university setting. This should be the focus of future studies to describe these pathways in more detail. Nevertheless, the presented findings confirm that the UFair Questionnaire is a valid tool for universities to assess the stress risk of their students and to identify aspects that should be improved to reduce the mental health burden and enhance the mental well-being of their students.

## 5. Conclusions

To measure perceived injustice among university students, we developed the so-called UFair Questionnaire based on previous literature and a qualitative study. The developed questionnaire consists of 20 items and showed very good psychometric properties. The questionnaire is available to interested scientists in German and English and can therefore be used and validated in future studies. Due to the differences in tertiary education between countries, it would be interesting to use the UFair questionnaire in other countries for data comparison. This will help to identify the global relevance of university fairness and to identify potential differences between diverse institutions and cultural settings.

Our findings underline that organizational injustice exists in the university setting, and that perceived injustice is associated with various mental health outcomes. This knowledge is important for prevention and health promotion in universities. It seems important to sensitize lecturers, administrative staff, and students to this topic. The reduction of potential sources of injustice may help students to stay healthy during studies and start a healthy working life.

## Figures and Tables

**Table 1:** Mean values, standard deviation (SD), internal consistency, and exploratory factor analysis (EFA) of the UFair Questionnaire items.

			Descriptive		Internal Consistency		EFA
	Item German	Item English	Mean	SD	Item-Total Correlation	α If Item Deleted	Factor Loadings λ
1	Die Notenvergabe an meiner Hochschule empfinde ich als ungerecht.	I experience the grading system at my university as unfair.	2.64	1.209	0.746	0.958	0.775
2	Die Vergabe von Themen für Referate, Haus- oder Abschlussarbeiten an meiner Hochschule empfinde ich als ungerecht.	I experience the assignment of topics for presentations, seminar papers or theses at my university as unfair.	2.48	1.216	0.736	0.958	0.766
3	Ich empfinde die Zulassungsverfahren für Lehrveranstaltungen an meiner Hochschule als ungerecht.	I experience the procedures for course admission at my university as unfair.	2.52	1.240	0.695	0.959	0.727
4	Ich empfinde, dass die Verfahren und Abläufe an meiner Hochschule von persönlicher Voreingenommenheit und Bevorzugung geprägt sind.	I experience the procedures and processes at my university as characterized by personal bias and favoritism.	2.62	1.219	0.740	0.958	0.769
5	Ich empfinde, dass Anliegen an meiner Hochschule schwer adressierbar sind oder nicht zeitnah bearbeitet werden.	I experience that concerns at my university are difficult to address or are not contemporary processed.	2.87	1.171	0.674	0.959	0.705
6	An meiner Hochschule empfinde ich die Möglichkeit von Studierenden, sich an wichtigen Entscheidungen zu beteiligen, als eingeschränkt.	I experience the opportunity for students to participate in important decisions as limited at my university.	2.91	1.142	0.650	0.959	0.682
7	Ich fühle mich von meiner Hochschule nicht zeitnah und umfassend informiert.	I do not feel that my university informs me promptly and comprehensively.	2.78	1.220	0.678	0.959	0.709
8	Ich empfinde, dass Änderungen an meiner Hochschule nicht transparent kommuniziert werden.	I experience changes at my university not to be communicated transparently.	2.81	1.209	0.679	0.959	0.710
9	An meiner Hochschule gibt es keine Möglichkeit, Beschwerde einzureichen.	There is no possibility to file a complaint at my university.	2.41	1.250	0.681	0.959	0.714
10	Dozierende an meiner Hochschule stellen wichtige Informationen nicht zeitnah bereit.	Lecturers at my university do not provide important information in a timely manner.	2.60	1.141	0.731	0.958	0.761
11	Erwartungen werden von den Dozierenden an meiner Hochschule nicht klar kommuniziert.	Expectations are not clearly communicated by the lecturers at my university.	2.67	1.155	0.736	0.958	0.766
12	Dozierende an meiner Hochschule behandeln Studierende respektlos.	Lecturers at my university treat students disrespectfully.	2.20	1.190	0.771	0.958	0.801
13	Ich empfinde die Dozierenden an meiner Hochschule als unaufrichtig.	I experience the lecturers at my university as insincere.	2.32	1.158	0.784	0.957	0.813
14	Ich habe oft das Gefühl, dass ich an meiner Hochschule ungerecht behandelt werde.	I often feel treated unfairly at my university.	2.28	1.219	0.794	0.957	0.821
15	Ich empfinde, dass das Verhalten von Dozierenden an meiner Hochschule von persönlichen Vorurteilen geprägt ist.	I experience the behavior of lecturers at my university as characterized by personal prejudices.	2.49	1.196	0.772	0.958	0.802
16	Ich empfinde, dass die Dozierenden an meiner Hochschule bestimmte Studierende bevorzugen.	I experience that the lecturers at my university favor certain students.	2.56	1.183	0.750	0.958	0.781
17	Dozierende bieten keine Möglichkeit, den eigenen Standpunkt zu vertreten.	Lecturers do not provide the opportunity to defend one’s own point of view.	2.49	1.150	0.750	0.958	0.780
18	Dozierende an meiner Hochschule sind für Kritik nicht offen.	Lecturers at my university are not open to criticism.	2.63	1.140	0.721	0.958	0.754
19	Individuelle Lebensumstände der Studierenden werden an meiner Hochschule nicht berücksichtigt.	The individual circumstances of students are not considered at my university.	2.83	1.247	0.696	0.959	0.728
20	An meiner Hochschule werden Studierende aufgrund ihrer finanziellen Möglichkeiten benachteiligt.	Students at my university are disadvantaged because of their financial means.	2.49	1.237	0.698	0.958	0.730
	Cronbach’s a					0.960	
	Eigenvalue						11.42
	Percentages of variance						57.11

**Table 2:** Participant characteristics.

		% or Mean	n or S.D.
Gender			
	Female	50.14	554
	Male	49.86	551
Age (years)		25.51	5.43
Type of institution of higher education			
	University	67.24	743
	University of applied sciences	32.76	362
Alcohol consumption			
	Never	18.21	199
	Once a month or less	30.83	337
	Two to four times a month	34.22	374
	Two to three times a week	12.17	133
	Four or more times a week	4.57	50
Smoking habits			
	Yes, daily	10.49	115
	Yes, occasionally	14.78	162
	No, not anymore	14.14	155
	Never smoked	60.58	664
Physical activity			
	No physical activity	7.71	84
	Less than 1 h a week	19.28	210
	Regularly 1 to 2.5 h a week	37.47	408
	More than 2.5 h a week	35.54	387
Nutrition: number of portions of fruit and vegetables per day			
	0–2 portions	47.07	514
	2–4 portions	45.33	495
	5 or more portions	7.60	83
International student			
	Yes	15.51	168
	No	84.49	915
Subjective Social Status (SSS)			
	Low SSS	38.10	421
	Middle SSS	19.82	219
	High SSS	42.08	465

**Table 3:** Mean values of the UFair Questionnaire and group differences for international students and SSS subgroups.

		Mean	SD	N	Test Value	*p* Value	Effect Size ^a^
Total		2.58	0.89	1097			
International students					7.796	<0.001	0.856
	Yes	3.14	1.07	168			
	No	2.47	0.81	911			
Subjective Social Status (SSS)					2.578	0.076	0.005
	Low SSS	2.59	0.81	416			
	Middle SSS	2.46	0.83	218			
	High SSS	2.62	0.97	463			

^a^ Cohen’s d in T-test and Eta-squared in ANOVA.

**Table 4:** Linear regressions of the UFair Questionnaire on depression or burnout for the total sample, international students, and SSS subgroups.

	Model I					Model II				
	B	Std. Error	Beta	*p*-Value	R^2^	B	Std. Error	Beta	*p*-Value	R^2^
**Depression**										
Total	0.376	0.022	0.473	<0.001	0.224	0.386	0.022	0.485	<0.001	0.261
Inter. students	0.539	0.039	0.735	<0.001	0.540	0.523	0.041	0.713	<0.001	0.579
Not inter. students	0.328	0.026	0.388	<0.001	0.150	0.347	0.027	0.410	<0.001	0.189
Low SSS	0.299	0.039	0.365	<0.001	0.133	0.313	0.040	0.382	<0.001	0.167
Middle SSS	0.284	0.048	0.377	<0.001	0.142	0.278	0.052	0.370	<0.001	0.192
High SSS	0.453	0.030	0.580	<0.001	0.336	0.468	0.032	0.598	<0.001	0.371
**Burnout**										
Total	0.554	0.032	0.467	<0.001	0.218	0.570	0.033	0.481	<0.001	0.250
Inter. students	0.507	0.054	0.598	<0.001	0.357	0.464	0.054	0.547	<0.001	0.439
Not inter. students	0.595	0.040	0.447	<0.001	0.200	0.619	0.041	0.465	<0.001	0.231
Low SSS	0.511	0.058	0.402	<0.001	0.162	0.539	0.060	0.424	<0.001	0.193
Middle SSS	0.512	0.066	0.473	<0.001	0.224	0.529	0.072	0.488	<0.001	0.244
High SSS	0.602	0.045	0.538	<0.001	0.289	0.599	0.047	0.535	<0.001	0.311

Model I: unadjusted. Model II: adjusted for age, gender, type of institution of higher education, and lifestyle factors (alcohol consumption, smoking, physical activity, nutrition).

## Data Availability

The datasets generated and analyzed during the current study are not publicly available due to German data protection regulations and the assurances in the informed consent agreement and ethic approval that the data will not be disclosed, but are available from the corresponding author on reasonable request.

## Abbreviations

The following abbreviations are used in this manuscript:

ANOVA	Analysis of Variance
CFA	Confirmatory Factor Analysis
CFI	Confirmatory Fit Intex
EFA	Exploratory Factor Analysis
ERI	Effort–Reward Imbalance
KMO	Kaiser–Meyer–Olkin
MBI-SS	Maslach Burnout Inventory for Students
PHQ-8	Patient Health Questionnaire
SD	Standard Deviation
SSS	Subjective Social Status
TLI	Tucker–Lewis Index
UFair	University Fairness Questionnaire
